# Structured Walking and Chronic Institutionalized Schizophrenia Inmates: A pilot RCT Study on Quality of Life

**DOI:** 10.5539/gjhs.v8n1p238

**Published:** 2015-05-20

**Authors:** Siew Yim Loh, Amalina Abdullah, Abdul Kadir Abu Bakar, Maniam Thambu, Nik Ruzyanei Nik Jaafar

**Affiliations:** 1 Department of Rehabilitation Medicine, Faculty of Medicine, University of Malaya Kuala Lumpur, Malaysia; 2 Department of Psychiatry. Hospital Permai. Johore, Malaysia; 3 Department of Psychiatry, Universiti Kebangsaan Malaysia Medical Centre, Malaysia

**Keywords:** walking, chronic schizophrenia, inactivity, psychiatric symptoms, quality of life, PANSS, Positive and Negative Syndrome Scale, PSP, Personal and social performanc

## Abstract

**Background:**

Lifestyle moderate-intensity physical activity can lower the risk of over twenty chronic health conditions, whilst inactivity reduces daily functioning and physical health of individuals living with schizophrenia. This study conducted in 2014 examines the effect of structured walking participation on QOL, psychosocial functioning and symptoms in Hospital Permai, one of the largest psychiatry institution in Asia

**Method:**

Chronic patients with schizophrenia (n=104) who met inclusion criteria were randomised to either a 3-month structured walking intervention or a treatment-as-usual arm. The Positive and Negative Syndrome Scale (PANSS), global functioning (PSP) and QOL (SF-36) were measured at baseline and after the 3-month interval.

**Results:**

At 3 month follow-up, there were significant within group differences in QOL (SF-36), psychiatric symptoms (PANSS), and personal and social performance (PSP). There were statistically significant increase in the median SF-36 scores, with increases shown in physical functioning (p<.001), physical role limitations (p<.05), social functioning (p<.01) in the intervention group compared to treatment-as-usual group. Statistically significant reduction of median PANSS score of the intervention group were noted in positive (p<0.001) and negative (p<0.01) symptom, and general psychopathology (p<0.01) scales. Statistically significant increase in the median PSP score (p<0.01) was found in the intervention group compared with the treatment-as-usual group. Between-group differences at post intervention (favouring Intervention) were significant for PANSS positive and SF36 Physical

**Conclusion:**

In long stayed chronic inmates, a simple but consistent, organized walking intervention has the potential to bring improvement in functioning, reduction in psychiatric symptoms and quality of Life. The emphasis of rehabilitation should target at lifestyle redesign intervention.

## 1. Introduction

Schizophrenia is a debilitating mental illness characterized by poor occupational and social functioning (vans Os, & Kapur, 2009), with a lifetime prevalence and incidence rate at 0·30–0·66% and 10·2–22·0 per 100 000 person-years respectively (McGrath, Saha, Chant, & Welham, 2008). Asian countries such as Hong Kong, Taiwan, and Korea have a lower lifetime prevalence rates (0.1 to 0.4 per 100 persons) (McGrath, Saha, Chant, & Welham, 2008; Chen, Wong, Lee, Chan-Ho, Lau, & Fung, 1993; Hwu, Yeh, & Chang, 1989). In contrast, non-Asian countries such as the US, New Zealand and Finland are estimated at between 0.3 and 1.6 per 100 persons (McGrath, Saha, Chant, & Welham, 2008; Kendler, Gallagher, Abelson, & Kessler, 1996; Lehtinen, Joukamaa, Lahtela, Raitasalo, Jyrkinen, Maatela, & Aromaa, 1990). In Malaysia, the incidence rate of schizophrenia is 5 per 100, 000 in a year (Chee et al., 2012; Aziz et al., 2008).

Many long-stayed patients developed co-morbidities after admissions to mental institutions. Mohammad, Tan and Gill (2010) reported that 85/134 (63.4%) institutionalized subjects had hyperlipidemia (24.6%), hypertension (12.7%) and diabetes mellitus (12.7%). In developing countries, such as China and Japan, patients are mostly deinstitutionalized as a result of limited beds in psychiatric hospital beds. About 90% of the 4.8 million individuals with schizophrenia stay with their family or significant others (Phillips, 1993). In Japan, deinstitutionalization of long-stay patients with schizophrenia showed improvement in their positive and negative symptoms, work and social functioning (Ryu et al., 2006). However, in Malaysia, a trend to institutionalize is observed and could further aggravate the burden of care. Patients end up staying for decades in the mega-institution due to the poor support from the families and the community (Mohammad, Tan, & Gill, 2010). In the Permai Hospital, where participants for the present study were recruited, the long stayed inmates were recently relocated to a much larger institution, with a capacity of about 1000 bedded.

A key dysfunction in schizophrenia is impairments of personal functioning, i.e. simple and complex activities of daily living (ADLs), and skills in jobs and housework, and social functioning (Strassnig, Signorile, Gonzalez, & Harvey, 2014; Harvey, 2010) due to multifactor ranging from chronic psychiatric (both positive and negative) symptoms, low economic background and lack of integrated physical and mental healthcare services (Kemp, Bates, & Isaac, 2009; Strassnig, Brar, & Ganguli, 2006). Strassnig, Signorile, Gonzalez, and Harvey (2014) argued that improving functioning of people with schizophrenia by organizing structured physical activity programs may reduce disability, improving ADLs, global functioning, symptoms and cognitions in schizophrenia. Sedentary lifestyle is a major public health problem in these long stayed inmates. Despite beneficial impact of a physical activity intervention have been reported for weight reduction, BMI, cholesterol, negative symptoms, as well as increasing mental health, cognitive, and global functioning (Vancampfort et al., 2012; Methapatara & Srisurapanont, 2011; Attux, Martini, Araújo, Roma, Reis, & Bressan, 2011; Van Citters, Pratt, Jue, Williams, Miller, Xie, & Bartels, 2010; Acil, Dogan, & Dogan, 2008; Duraiswamy, Thirthalli, Nagendra, & Gangadhar, 2007; Poulin et al., 2007), many inmates are sedentary. In tandem with the World Mental Health promotion celebrated in the month of October, with an emphasis on schizophrenia, the needs for further research is warranted. In chronic schizophrenia, in the field of physical activity and quality of life (QOL), there have been controversial results (Barnes et al., 2012; Fleury et al., 2013). As an example, an exercise intervention on chronic patients with schizophrenia, showed improved short-term memory (Pajonk et al., 2010) but with no improvement in positive and negative symptoms (Heggelund, Nilsberg, Hoff, Morken, & Helgerud, 2011). More lifestyle intervention is warranted for individuals with chronic schizophrenia whose unremitting illness may produce even poorer physical and daily functioning. Therefore, this present study will be the first to look at the efficacy of an organized “walking” (as a potent lifestyle activity) intervention in chronic, institutionalized schizophrenia patients in Malaysia.

## 2. Methods

### 2.1 Study Design

This is a 2-arm randomized controlled trial (RCT) design. Allocation ratio of participants between intervention and treatment-as-usual groups was 1:1. The independent variable of the study is the intervention (structured walking program) vs treatment-as-usual groups. The dependent variables of the study are QOL (SF-36), psychiatric symptoms (PANSS), and personal and social performance (PSP). A follow-up was conducted after completion of the 3-month organized walking intervention.

### 2.2 Participants

A 3-month program aiming to increase physical activity in chronic, long-stay schizophrenia patients was implemented at the Permai Institute in Johor Bahru, Malaysia. The multidisciplinary program team consisted of a group of professionals from diverse disciplines (therapists, nurses, psychiatrists, nutritionist, and pharmacist). Diagnoses were based on the patient edition of the Structured Clinical Interview for DSM-IV Axis I Disorders 31 conducted by trained senior psychiatrists. The inclusion criteria for the study included adult patients with schizophrenia between the age of 18 to 65 years old, diagnosis of schizophrenia based on clinical interview using DSM-IV criteria, patients receiving inpatient treatment from Hospital Permai Johor Bahru, and patients who are able to understand the information sheet, questionnaires and consent procedure. Exclusion criteria included bedridden patients or patients with acute medical illnesses such as acute coronary syndrome, acute respiratory illness, stroke, limb pain or fractures, patients with acute psychotic symptoms or in relapse, and patients diagnosed with dementia.

Eligible participants were informed that they may choose to drop-out of the study at any given time, without reprimand. Informed consent was taken from all participants by the researcher who was not involved in conducting primary treatment of patients to minimize bias. Participants were screened for any cardiac, neurological or locomotors problems through review of medical history, physical examination and ECG, to ensure safe participation in this study. Participants who were discharged before the end of the study were considered as drop-outs. Suspension of study would occur if any participants experienced physical harm as a result of the organized walking intervention.

### 2.3 Randomization and Blinding

Participants were randomly assigned to either the intervention or control (treatment-as-usual) groups using a computerized random number generator. Randomization was done in blocks of two to ensure equal number of patients in each group. Allocation of participants were concealed from the primary investigators of the study as randomization codes were kept by a research assistant not involved in this study and further concealed by sealing codes in envelopes.

### 2.4 Treatment Procedure

**Treatment arm:**

A 3-month, group organized walking exercise was chosen as the lifestyle intervention physical activity, being also the simplest form for these long stayed inmates. Given that participants would be inactive at the start of the study, the walking exercise was graded as 3-times per week regime; with gradual increase in duration of sessions. In the first month, participants partake in a 20-minute walking exercise per session with 5-minute warm-up and 5-minute warm down sessions. In the second month, the session increased to 30-minutes walking exercise per session with 5-minute warm-up and 5-minute warm down sessions. In the third month, the session increased to 40-minute walking exercise with 5-minute warm-up and 5-minute warm down sessions. The walking exercises were conducted within the vicinity of the vast compound of the institution. Participants were supervised during the exercise by ward staff nurses and assistant medical officers. Participants’ pulse rate was monitored before and after the exercise to prevent overexertion during exercise.

**Control arm:**

Subjects in the treatment-as-usual group will continue their usual daily activities, plus their usual pharmacological treatment and nursing care from the hospital. They were allowed to work, go for outing and join other ward activities. Some of the participants in the treatment-as-usual group perform their own light exercises as part of their daily activities such as walking to the canteen or around the ward area and gardening.

### 2.5 Measures

The questionnaire and data collection was administered through face-to-face interviews with participants. Any additional information needed regarding the participants was obtained through interview with ward staff members. Training of the method in administering questionnaires (IPAQ-M, SF-36, PSP and PANSS) to participants was provided by a trained, senior psychiatrist. The socio-demographic questionnaires and the IPAQ-M were measured at baseline. Outcome measures SF-36, PSP and PANSS were measured both at baseline and at 3-month follow-up.

**Baseline Measures**

Socio-Demographics Questionnaire

Demographic data collected include age, gender, ethnicity, marital status, smoking status, education level, employment status, height, weight, BMI and waist circumference, duration of illnesses, length of ward stay, co-morbid illnesses, and medication (both somatic and psychiatric).

International Physical Activity Questionnaire, Malay version (IPAQ-M)

The IPAQ-M (Chu, & Moy, 2012) was used to assess the level of physical activity of the subjects prior to the start of the study. It is a 27 item, self-administered questionnaire. IPAQ-M assesses physical activity across comprehensive set of domains including leisure time physical activity, domestic and gardening activities, work-related physical activity, and transport-related physical activity. Chu and Moy (2012) has examined the reliability and validity of the Malay version of IPAQ (IPAQ-M) in 81 Malay adult participants. The intra class correlation coefficient (ICC) scores revealed moderate to good correlations (ICC = 0.54-0.92; p<.001) on items categorized by intensities and domains and a kappa, κ of 0.73 for total activity. Validity results from the PA-Log were statistically significant (p<.001) across intensities and domains (ρ = 0.67-0.98). The IPAQ-M demonstrated good reliability and validity for the assessment of physical activity among this population.

**Primary Outcome**

36-item Short-form Survey (SF-36)

The SF-36 (Ware, & Sherboume, 1992) is a questionnaire measuring health-related quality of life (HRQOL). The questionnaire has good internal reliability (Cronbach’s alpha >0.85) and construct validity in terms of distinguishing health differences between groups (Sararaks et al., 2005). In 2005, the UK English version of the SF-36 was translated to the Malay language under the aegis of International Quality of Life Assessment (IQOLA). The SF-36 includes one multi-item scale that assesses eight health concepts and they include physical functioning, role limitations due to physical problems, vitality, bodily pain, social functioning, and role limitations due to emotional problems, mental health, and general health. In addition, the questionnaire also provides a single item for perceived change in health. Items are scored and aggregated to provide a scale ranging from 0-100 (0= poor health and 100= good health). Higher scores indicate a better health-related quality of life.

**Secondary Outcomes**

Positive and Negative Syndrome Scale (PANSS) for Schizophrenia

PANSS (Kay, Flszbein, & Opfer 1987) is an instrument that provides a balanced representation of positive and negative symptoms of schizophrenia, and gauges their relationship to one another as well as to global psychopathology. It is a 30-item, 7-point rating scale with each rating scale accompanied by a complete definition that represent increasing levels of psychopathology: 1=absent, 2=minimal, 3=mild, 4=moderate, 5=moderate-severe, 6=severe, and 7=extreme. The PANSS is scored by summation of ratings across items, such that the potential ranges are 7-49 for the Positive and Negative Scales and 16-112 for the General Psychopathology Scale. In addition to the three scales, a bipolar Composite Scale was calculated to conceive the degree of difference and direction between positive and negative symptoms in order to gauge the level of predominance between the two symptoms. The Composite Scale is calculated by subtracting the negative from positive score, thus yielding a bipolar index that ranges from -42 to +42. Kay et al. (1987) reported that a study of 101 schizophrenics found the four scales to be normally distributed and supported their reliability and stability. Kay et al. (1987) reviewed 5 studies that showed evidence of PANSS having good criterion-related validity, predictive validity, drug sensitivity, and utility for both typological and dimensional assessment.

Personal and Social Performance (PSP) Scale

The PSP scale (Morosini, Magliano, Brambilla, Ugolini, & Pioli, 2000) is a reliable and quick measure of global functioning in patients with mental illnesses. The scale is a 100-point single item measure, subdivided into 10-point categories. It assesses personal and social functioning in terms of four main areas: socially useful activities, personal and social relationships, self-care, disturbing and aggressive behaviors. The four areas are assessed separately on a 6-category severity scale which ranges from “absent” to “very severe”. Based on the severity scores of the four areas mentioned, a 10-point category is selected. A total score of 100-91 reflects excellent functioning; a score of 90-81 reflects good functioning; 80-71 reflects mild difficulties; 70-61 reflects manifest but not marked difficulties in one or more area; 60-31 reflects marked to severe difficulties; 30-0 reflects poor functioning requiring intensive support or supervision. The scale has been found to have good reliability with a weighted kappa of 0.94 and ICC = 0.94.

### 2.6 Sample Size Calculation

Sample size calculation was conducted using G*Power based on data collected from a similar study by Acil, Dogan, and Dogan (2008). The effect size for the difference in the physical domain of QOL (WHOQOL-BREF-TR) scale score at the end of 10-weeks in the study was 0.47 (medium-to-large). To achieve 80% power, and a significant power α=0.05 the total sample size needed for both groups is 116 (58 participant in each group). In this study, number of participants recruited was 104 due to time constraints and a large number of patients who did not meet the inclusion criteria.

### 2.7 Data Analysis

Data were analysed using SPSS version 21 software. Analyses were conducted using the intention-to-treat analysis. Normality tests were performed using the Kolmogorov-Smirnov method, where data was found to be not normally distributed. Further analysis was performed using non-parametric tests. Comparison of baseline measures scores between the groups were conducted using Chi-square tests and Mann-Whitney U tests to determine if baseline measures were comparable between the groups. Wilcoxon signed ranks test was used to compare median scores of SF-36, PSP and PANSS between participants in the intervention and treatment-as-usual groups at baseline and 3 months follow-up.

## 3. Results

### 3.1 Baseline and Clinical Characteristics

**Figure 1:**
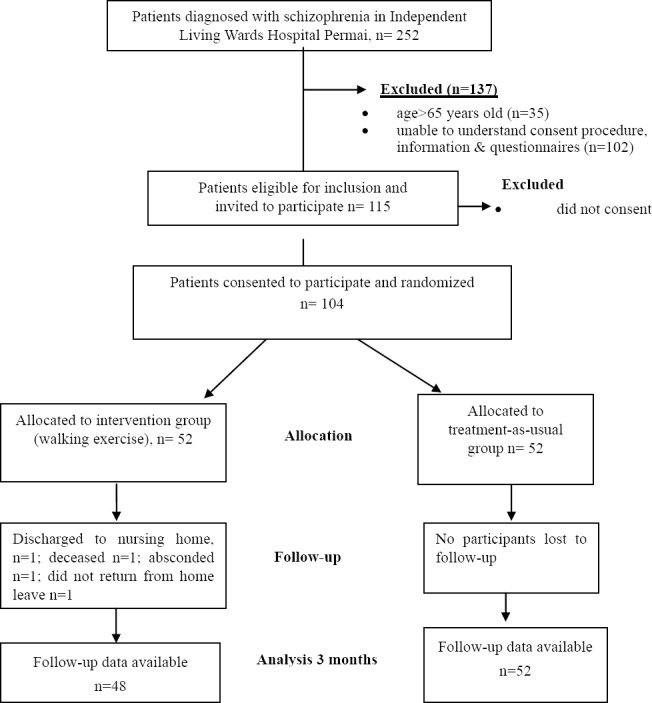
Participant Flow Diagram

There were a total of 252 patients recruited between July to September 2013 from the Independent Living Wards, Hospital Permai. Out of this number, 137 patients were excluded because they were above 65 years old, or were unable to understand the information sheet and questionnaires. 115 patients were eligible for participation in the study, however a further 11 patients declined to participate. Hence, the final number of patients who consented and participated in the full procedure of the study was 104 participants. Participants were then randomly assigned to either the organized walking regime (intervention) (N=52) or treatment-as-usual (control) group (N=52). In the intervention group, four (7.8%) participants had dropped out of the study, due to the follow reasons: discharged to nursing home, deceased, absconded, and did not return from home leave. Figure 1 shows the participant flow diagram.

The participants’ age ranged from 23 to 65 years old (M=21.6, SD=10.19). Duration of ward stay was between 0.4 to 49 years (M=9.43, SD=13.18). Majority of the final sample were male patients (71.2%). Malay participants made up the majority of the ethnic group in the sample (61.5%) followed by Chinese (29.8%), and Indian (8.7%). Most of the participants are single (75%), non-smokers (51%), and secondary level education (60.6%). There were equal number of participants that were employed and unemployed at baseline. Mean of participants weight at baseline was 67.06 kg (SD=14.44), BMI at baseline was 25.1 (SD=4.86), and waist circumference at baseline was 93.92 (SD=13.18). Table 1 shows the comparison of baseline (socio-demographic) and clinical characteristics between participants in intervention group and treatment-as-usual group.

**Table 1:** Comparisons of socio-demographic and clinical characteristics between participants in intervention and treatment-as-usual group

	Intervention (n=52)		Treatment-as-usual (n=52)		p-value
	
	n	%	n	%	
***Gender***					**0.38**
Male	35	67.3	39	75.0	
Female	17	32.7	13	25.0	
*Ethnicity*					0.01
Malay	39	75.0	25	48.1	
Chinese	9	17.3	22	42.3	
Indian	4	7.7	5	9.6	
***Marital status***					**0.13**
Single	41	78.8	37	71.2	
Married	6	11.5	13	25.0	
Divorced	5	9.6	2	3.8	
***Cigarette***					**0.08**
Smoker	30	57.7	21	40.4	
Non-smoker	22	42.3	31	59.6	
***Education***					0.015
No formal	2	3.8	7	13.5	
Primary	10	19.2	19	36.5	
Secondary	37	71.2	26	50.0	
Tertiary	3	5.8	0	0.0	
***Physical* activity**					**0.06**
Low	18	34.6	34	65.4	
Moderate	14	26.9	10	19.2	
High	20	38.5	8	15.4	
*Age	46.0 (14)		53.00 (11)		<0.01
*BMI	24.9 (6.9)		23.93 (7.1)		0.10
*Waist circumference	95.5 (16.8)		89.00 (18.5)		0.04
*Duration of illness	15.5 (12)		25.00 (12)		<0.01
*Duration of ward stay	6.0 (8)		9.00 (14)		0.08

### 3.2 Primary Outcome

**Comparisons in QOL Scores between Groups at Baseline and Follow-up**

A Wilcoxon signed ranks test was used to investigate QOL scores between groups at baseline and follow-up. In the intervention group, there were statistically significant increased median SF-36 scores at follow-up, in the components of physical functioning (p<0.01); physical role limitations (p=<0.04); and social functioning (p= 0.07). The physical component score in the intervention group also increased at follow-up, and this is also statistically significant (p<0.01). In the treatment-as-usual group, the same SF-36 component scores were approximately the same at follow-up as compared to baseline; and this is not statistically significant. Table 2 shows a comparison of quality of life (SF-36) scores in the intervention group and treatment as-usual-group, at baseline and 3-months follow-up.

**Table 2:** Differences in SF-36 scores between participants in intervention and treatment-as-usual groups

SF-36 domain	Intervention			Treatment-as-usual		
	
	Baseline median (IQR) n = 52	3 months median (IQR) n = 48	p-value*	Baseline median (IQR) n = 52	3 months Median (IQR) n = 48	p-value*
Physical functioning	67.51 (33.75)	90.00 (27.50)	<0.01	95.0 (15.0)	95.0 (10.0)	0.89
Physical role limitations	68.75 (48.44)	81.25 (43.75)	0.04	100.0 (17.19)	100.0 (17.19)	0.59
Bodily pain	62.00 (48.00)	80.00 (38.00)	0.06	100.0 (38.0)	100.0 (38.0)	0.89
General health	63.50 (25.00)	69.50 (34.50)	0.15	67.00 (27.0)	65.0 (28.0)	0.32
Vitality	56.25 (23.44)	56.25 (25.00)	0.99	56.25 (18.75)	56.2 (12.5)	0.34
Social functioning	75.00 (43.75)	87.50 (34.38)	0.01	100.0 (25.0)	100 (12.5)	0.32
Emotional role limitations	70.84 (47.92)	79.17 (50.00)	0.23	100.0 (0.0)	100.0 (6.25)	0.64
Mental health	70.00 (25.00)	65.00 (28.75)	0.13	70.0 (15.0)	70.0 (10.0)	0.01
Physical component score	49.28 (11.01)	52.91 (7.47)	<0.01	56.0 (7.5)	55.8 (9.98)	0.58
Mental component score	48.49 (14.56)	47.20 (14.63)	0.63	50.5 (6.41)	51.0 (4.48)	0.29

* *Note.* Wilcoxon Signed Ranks Test;

IQR= Inter-quartile range.

### 3.3 Secondary Outcomes

**Comparison of PSP and PANSS scores between Groups at Baseline and Follow-up**

A Wilcoxon signed ranks test was used to compare PSP and PANSS scores between the groups, at baseline and follow-up. In the intervention group, median PSP score was higher at follow-up and this is statistically significant (<0.01). Median PSP score of participants in the treatment-as-usual group was the same at follow-up (p= 0.068) but it is not statistically significant. There was statistically significant reduction of median PANSS scores in the domain of positive (p<0.01), negative (p<0.01) and general psychopathology (<0.01) symptoms in the intervention group at follow-up. There was statistically significant reduction in the median PANSS score - general psychopathology scale (p<0.01) in the treatment-as-usual group at follow-up; although the median score of this group at follow-up was slightly higher than the intervention group at follow-up. Median PSP and PANSS positive scale scores in the treatment-as-usual group did not differ at follow-up compared to baseline. There was a slight reduction in the median PANSS negative scale score at follow-up in the treatment-as-usual group; however this was not statistically significant. Table 3 shows comparison of PSP and PANSS scores in the intervention and treatment-as-usual groups at baseline and 3 months follow-up are described.

**Table 3:** Differences in PSP and PANSS scores between participants in intervention and treatment-as-usual groups

	Intervention			Treatment-as-usual		
	
	Baseline median (IQR) n = 52	3 months median (IQR) n = 48	p-value*	Baseline median (IQR) n = 52	3 months median (IQR) n = 52	p-value*
PSP	75.5 (6.5)	77.0 (10.0)	<0.01	72.0 (11.0)	72.0 (10.0)	0.06
PANSS positive scale	8.0 (3.0)	7.0 (1.0)	<0.01	7.0 (1.0)	7.0 (1.0)	0.01
PANSS negative scale	9.0 (3.75)	8.0 (2.0)	<0.01	11.0 (8.0)	10.5 (6.0)	0.10
PANSS general psychopathology scale	21.0 (3.75)	17.0 (3.0)	<0.01	21.0 (5.0)	19.5 (2.0)	<0.01
PANSS composite scale	-1.0 (3.75)	0.0 (2.0)	0.55	-3.0 (11.0)	-3.0 (10)	0.68

* *Note.* Wilcoxon Signed Ranks Test;

IQR = Inter-quartile range, PSP = Personal and Social Performance Scale, PANSS = Positive and Negative Syndrome Scale.

## 4. Discussion

The results showed marked improvements in QOL at 3-month follow up scores in the components of physical functioning (p<.001), physical role limitations (p<.05), social functioning (p<0.01) in the intervention group compared to treatment-as-usual group. The improvements in QOL scores after exercise therapy have also been noted in previous studies. For example, Acil, Dogan, and Dogan (2008) showed significant improvement of QOL in participants who were adherent to a 10-week period of aerobic exercise regime as compared to the control group, assessed by the World Health Organization Quality of Life Scale-Turkish version. Furthermore, recent studies (Gomes, Bastos, Probst, Riberiro, Silva, & Corredeira, 2014; Vancampfort, Probst, Scheewe, Maurissen, Sweers, Knapen, & Hert, 2011) have found further evidence that adherence to an exercise intervention is related to improvement in QOL.

Statistically significant increase in the median PSP score (p<0.01) was also found at follow-up in the intervention group compared with the treatment-as-usual group. Statistically significant reduction of median PANSS score of the intervention group were noted in positive (p<0.001) and negative (p=0.01) symptom, and general psychopathology (p<0.01). Acil, Dogan, and Dogan (2008) reported that subjects in the exercise group had reduction in the scale for the Assessment of Positive Symptoms, Scale for the Assessment of Negative Symptoms and the Brief Symptom Inventory. Another study by Scheewe et al. (2012) found that 2 hours of structured exercises weekly for 6 months, significantly decreased PANSS positive and PANSS negative scale scores compared with occupational therapy. Several studies have shown the positive impact of exercise intervention on functioning. Duraiswamy, Thirthalli, Nagendra, and Gangadhar (2007) had found an an overall significant (*p* <.05) improvement in social competence. Two other studies (Belcher, 1988; Mrazek & Hatlova, 1995) have found that an organized exercise regime an improvement in patient’s communication, and interaction skills with other patients and health staffs. Recently, Kimhy et al. (2014) have found a correlation between physical activity and improved ADL and daily functioning. Furthermore, just as the results from this study, Vancampfort, Probst, Scheewe, Knapen, De Herdt, and De Herdt (2012) found a positive correlation between exercise capacity and global functioning in individuals with schizophrenia.

The significant findings add to the literature on the efficacy of an exercise intervention (organized walking regime) towards chronic patients with schizophrenia. The results suggest that even in chronic cases, exercise interventions have the potential to improve outcomes. The intervention chosen (ie organized walking regime) has good feasibility in a real-life setting for improvement in QOL, well-being and psychiatric symptoms. A greater impetus for mental health services and policies to shift from institutionalizing patients which can lead to limitations to the patients’ ADLs, towards a self-management approach where patients may play an active role in their recovery, to be prepared for reintegration into society.

## 5. Limitations of Study and Future Directions

Firstly, patients are non-blinded to the intervention that they were receiving. Participation in the walking exercise intervention itself may have patients to have a stronger motivation to improve their physical and mental conditions in the intervention group due to more interaction hours with helpers. Future studies would need to control for these variables as part of their analysis. It is also not known from the study whether the positive impact of a walking intervention on chronic schizophrenia patients can be generalized to other types of physical activity interventions (such as yoga, aerobics training, high intensity training). Currently, there is lack of consensus on which kind exercise interventions are suitable for this population (Gorczynski & Faulkner, 2010; Vancampfort et al., 2010). Hence, future studies should look at comparing the impact of different types of physical activity interventions towards clinical outcomes and QOL. Although enumerators were trained for both groups, questionnaires administered through interviews with patients can promote social desirability, and mood biases in responses (Podsakoff, MacKenzie, Lee, & Podsakoff, 2003). In addition to that, no objective measures were implemented to measure physical activity levels in patients at baseline and follow-up. Accelerometer could be used in future studies, as to have accurate measurement of physical activity levels is needed to achieve full efficacy of the exercise regime given to patients (Lindamer et al., 2008). Future studies need to implement objective measures such as with the use of a pedometer to measure physical activity levels. Future prospective studies should also be implemented to examine whether the observed effect is transient or long lasting. Lastly, the results of this study merit further investigation of neurobiological mechanisms underlying the therapeutic effect of exercise interventions should be conducted to understand the mechanism behind its effectiveness.

## 6. Conclusion

In conclusion, we demonstrated that walking participation had a positive effect on QOL, well being and psychiatric symptoms in individuals with chronic schizophrenia. Sedentary lifestyle of people living with chronic Schizophrenia in long term institution is a public health burden. This pilot study showed early evidence of the efficacy of a daily lifestyle physical activity (i.e walking) which should be further pursued for even the most debilitated, chronic schizophrenia patients. There is a need for mental health services to shift from institutionalizing to community housing and such simple lifestyle intervention (using a patient self-management approach) can cost-effectively help in preparing people to be reintegrated back into the society.

**Terminology**

PSP -Personal and Social Performance Scale;

PANSS -Positive and Negative Syndrome Scale for Schizophrenia;

IPAQ-M -International Physical Activity Questionnaire, Malay version.
